# Conversion of imager-specific response to tissue phantom fluorescence into system of units-traceable units

**DOI:** 10.1117/1.JBO.27.7.074715

**Published:** 2022-05-12

**Authors:** Maritoni Litorja

**Affiliations:** National Institute of Standards and Technology, Sensor Science Division, Gaithersburg, Maryland, United States

**Keywords:** standards, fluorescence imaging, system of units-traceability, fluorescent tissue phantoms, fluorescence-guided imaging, calibration

## Abstract

**Significance:**

The fluorescence-guided imaging for surgical intervention community recognizes the need for performance standards for these imaging devices. Tissue phantoms are used to track an imager’s performance as a fluorescence detector, but imager-specific units are of limited utility.

**Aim:**

Tissue phantoms can be calibrated to be traceable to the international system of units (SI) and in turn be used to calibrate imagers such that fluorescence measurements can be reported in universally accepted units.

**Approach:**

The radiometry to convert imager-specific arbitrary digital counts to SI-traceable unit of watts is described in this paper.

**Results:**

An example of an imager calibration is included.

**Conclusions:**

Calibrated tissue phantoms become a tool for metrological traceability.

## 1 Introduction

As the number of fluorescent contrast agents and optical imagers in development increases, the number of contrast agent/imager pairs used in fluorescence-guided imaging (FGI) for surgical interventions will increase commensurately.^1^ These contrast agent/imager systems are complex devices, having a chemical component, optical hardware, software, and diverse measurement goals. Consequently, standardization is a challenge.^2^ An imager’s measurement of the fluorescence from a particular contrast agent in the target tissue or organ is of interest because this is a physical quantity that would provide information to many parties in the FGI community—the contrast agent manufacturer, the optical imaging developer, the regulator, and the end-user.

Fluorescent tissue phantoms have been developed to monitor the repeatability and reproducibility of an imager’s measurement of fluorescence. The imager typically provides the digital counts or digital numbers N, for every pixel i in response to the fluorescence from the tissue phantom for a specific set of optical parameters. Any changes to the optical system, such as using a different geometric configuration, lens f-stop, or distance to the sample, e.g., can yield different counts, which makes it difficult to evaluate the meaning of the changes. A way to quantify and attribute changes in the imager’s response is to have it measure the flux from a system of units (SI)-traceable tissue phantom *in situ*, preferably at least before each use. Measurements would then have physical meaning in the sense that optical units would be attached to the measurements.^3^

### 1.1 SI-Traceable Measurements

**Fig. 1:**
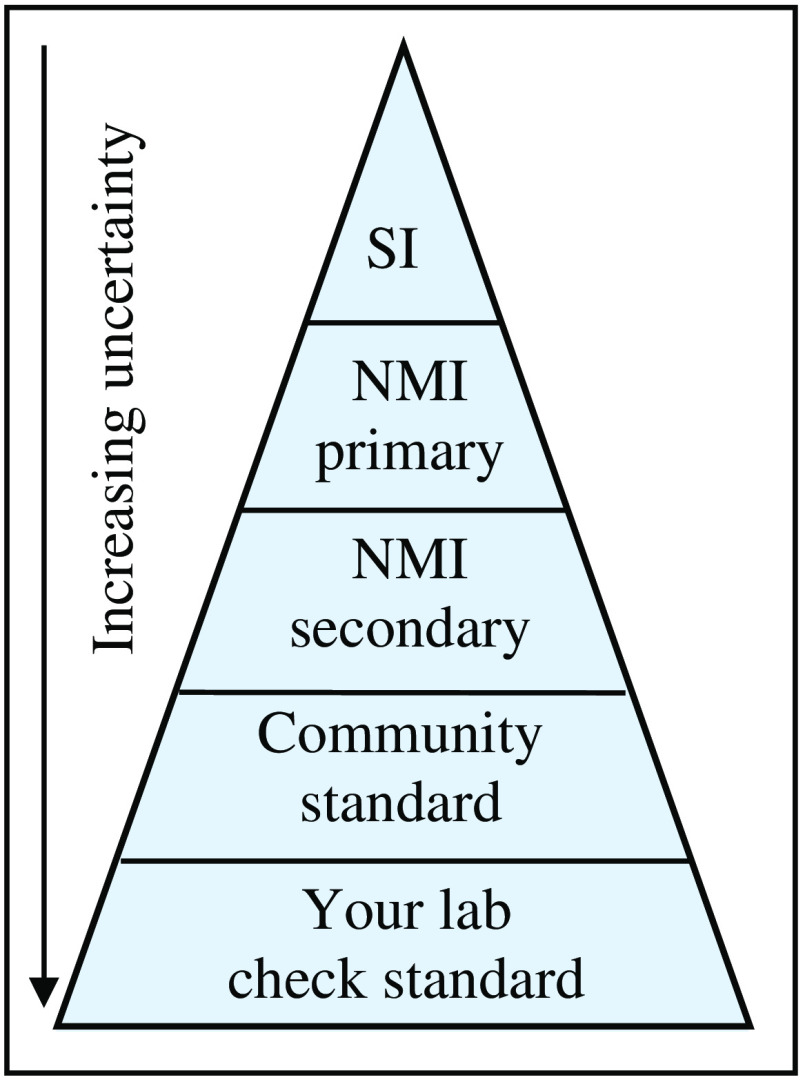
Illustration of the hierarchy of standards. The tissue phantoms can become a community standard.

The use of SI units enables measurement results to be compared across more than a single device and across time. For FGI, this is beneficial for situations in which different devices and configurations may be used to assess the effectiveness of a particular contrast agent; examples include comparing two different imaging devices, evaluating components, or quantifying the impact of changing collection parameters. SI-traceable units allow for physically tractable comparisons of device characteristics.^4^ The process of converting counts to a radiometric unit requires calibrating the imager using SI-traceable artifacts. SI traceability is formally defined as the “property of the result of a measurement or the value of a standard whereby it can be related to stated references, usually national or international standards, through an unbroken chain of comparisons all having stated uncertainties.”^5^ The references may be physical standards such as optical sources and detectors or chemical standards, which are referred to as reference materials.^6^ Figure 1 is an illustration of the hierarchy of steps needed to go from an SI unit to standards used by the community. Each of these steps corresponds to a link in what is known as a traceability chain. At the top of this chain are the SI units based on universal physical constants, followed by the primary standard of a National Metrology Institute (NMI) used to physically realize the SI unit; secondary standards, which can be numerous, transfer the primary scale to reference instruments and artifacts that are then used to disseminate the standard unit to various communities for their scientific and commercial applications. There are often several steps in a traceability chain, and as illustrated by the width of the base of each step in the hierarchy, the uncertainty increases with each step away from the primary reference. Standards are created through consensus by a measurement community. The NMI works with measurement communities and facilitates the establishment of metrological traceability to the SI, as the latter guarantees comparability and universal acceptance of measurement results.^7^ In the USA, the National Institute of Standards and Technology (NIST) is the NMI.

### 1.2 Fluorescence Imaging Tissue Phantoms

The specific function of the tissue phantom addressed in this paper is its use as a calibration optical radiation source to determine the response of a test imager to radiant flux in SI-traceable units. The FGI community has collectively produced various tissue phantoms for imaging system performance evaluation.^8^^–^^10^ These physical constructs consist of a fluorescent material homogeneously mixed in varying proportions with absorbers and scatterers in a polymeric matrix to mimic fluorescence from tissues injected with a contrast agent.^11^ The fluorophore in the tissue phantom is a more stable proxy of the contrast agent(s), which may not be shelf-stable or may not be able to be embedded in a solid matrix.^12^ The fluorescence emitted from the surface, into free space, and collected by the imager is dependent on the optical characteristics of the tissue phantom material composition and the incident excitation radiation. The tissue phantom for establishing SI-traceability is a higher-order working standard and needs to have its emission be predictable. Often relatively simple in its physical construction, it needs to have a flat surface, be non-specular, and have easily measurable areal dimensions. This contrasts with functional tissue phantoms intended to mimic the shape of tissues and can be physically complex structures with a different set of performance evaluation goals.^13^ The sum of counts from all pixels $N_{i}$ attributed to the fluorescence, designated here as S, can be correlated with either the amount of fluorophore embedded in the tissue phantom, expressed in mole or kilogram, or the radiant flux from the tissue phantom, expressed in milliwatt or photons per second. The two are different physical quantities that can be made SI-traceable with different calibration routes. The first one requires calibration using the distribution of known amounts of material (SI-traceable to mole or kilogram) in the phantom, whereas the second requires a calibrated optical radiation source, heretofore referred to as source (SI-traceable to the optical watt). The fluorophore in the tissue phantom is itself a proxy for the contrast agent to be used *in vivo*, so it may not be a chemically significant substance; what is useful is when the relationship between its fluorescence output to that of the contrast agent of interest can be established. This concept of equivalency^14^ is used by the flow cytometry community in which there are many types of fluorophores used, sometimes even together in a single bead, and many devices to detect them. The community has successfully established measurement standards^15^ and a quantitation consortium.^16^

A single tissue phantom may be used to validate the performance of an imager intended for various contrast agents, and thus it is convenient to use the optical radiation-based calibration pathway, especially because optical radiation is a direct measurand of an imager. Both SI-traceability pathways will ultimately be needed to establish the response of an imager to a specific contrast agent. For the optical imager alone, its performance as a light collector can be evaluated using the tissue phantom as a calibrated source.

## 2 Calibration of Tissue Phantoms as a Test Light Source

The goal of calibrating the imager in an FGI system is to determine how the reported counts correspond to the physical quantity of fluorescence radiant flux detected. Thus, a source of known radiance, with emission at the spectral band at which the imager is designed to detect, is needed. The tissue phantom can become a source of known radiance flux through calibration. Table 1 is a list of relevant radiometric quantities and associated units for calibration.

**Table 1:** Description of relevant radiometric quantities, symbols, and units.

Physical quantity	Symbol	Unit	Description
Radiant flux	Φ	mW	Total optical energy
Radiance	L	$\mathrm{mW}\;{\mathrm{cm}}^{-2}\;{\mathrm{sr}}^{-1}$	Radiant flux emitted by a surface per unit area per steradian, such as a source
Irradiance	E	$\mathrm{mW}\;{\mathrm{cm}}^{-2}$	Radiant flux received by a surface per unit area, such as the camera image plane
Solid angle	ω	sr	Solid angle of optical collection
Imager fluorescence band responsivity	$R_{f}$	$\text{counts }{\mathrm{mW}}^{-1}$	Responsivity of the imager for the specified fluorescence spectral band

### 2.1 Description of Calibration Procedure

**Fig. 2:**
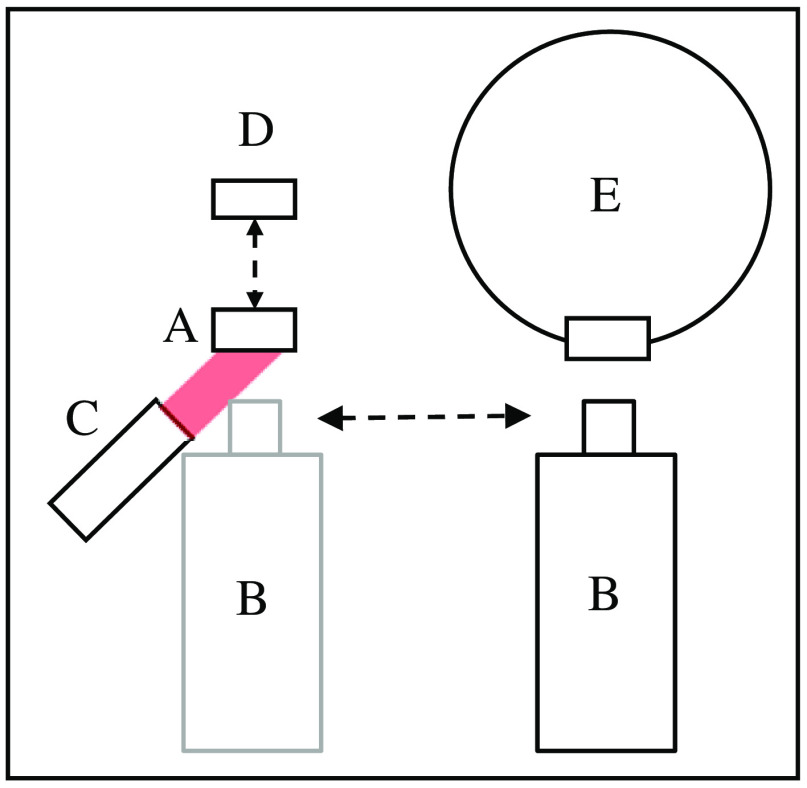
Experimental setup in the calibration of a tissue phantom (A) against a reference source (E) using a transfer camera (B). Irradiance from the excitation radiation beam (C) is measured using a photodiode (D).

The tissue phantom acquires its SI scale through comparison against a reference source. Because a reference source that matches the desired spectral band is not always available, one can be assembled and calibrated. The reference source needs to have an emission bandwidth less than the bandwidth of the FGI system for which the tissue phantom is designed. In practice, a narrowband source or a broadband source with a bandpass filter can be used. Figure 2 shows a diagram of the experimental setup of calibrating the tissue phantom against a reference source. The fluorescing tissue phantom (Quel Imaging, White River Junction, Vermont)^17^^,^^18^ (A) is imaged using a low noise transfer camera (Pixis 1024BR, Princeton Instruments), (B) fitted with an 800 nm long-pass filter (FEL0800, Thorlabs), an example of a filter used for FGI, whereas the tissue phantom is irradiated with a collimated beam of 780-nm radiation from an LED (M780L3, Thorlabs), (C) at an angle of incidence of 25 deg. The excitation irradiance at the plane of the tissue phantom is measured using a calibrated photodiode^19^ (S2281-04, Hamamatsu), (D) that is swapped in place of the tissue phantom before its image collection. The tissue phantom is then removed after its fluorescence image is taken and replaced with an integrating sphere source (IS-3, Thorlabs), (E) which serves as the reference source, described below in Sec. 2.2. An image of its exit port is taken. Both images are later processed for analysis. The radiant flux at the image plane $\Phi_{\text{image}}$ is a function of the radiance of the source $L_{\text{source}}$; the collection solid angle ω, which is calculated as the ratio of the lens aperture area to the square of the distance from source to the lens aperture; and the area of the source $A_{\text{source}}$ [Eq. (1)]. The camera’s response to the radiant flux is reported in counts for all pixels that correspond to the source being imaged. The sum of counts (N) for all pixels attributable (i) to the source fluorescence is recorded as S [Eq. (2)]. The imager’s responsivity in the specified spectral band, $R_{f}$, is the total counts S generated in response to $\Phi_{\text{image}}$ [Eq. (3)]

$$\Phi_{\text{image}}=L_{\text{source}}\cdot (\frac{A_{\mathrm{lens}\;\mathrm{apt}}}{d^{2}})\cdot A_{\text{source}},$$ (1)

$$S=\sum_{i}N,$$ (2)

$$R_{f}=\frac{S}{\Phi_{\text{image}}}.$$ (3)

In this radiometric calibration, it is implicit that the response of the transfer camera to the radiant flux remains the same when the tissue phantom and reference source are measured sequentially, as long as the test and reference generate the same level of response, all collection parameters are the same, and the measurements are made as close in time as feasible. In other words, the transfer camera’s $R_{f}$ (test) is equal to $R_{f}$ (ref) during the calibration. A tissue phantom test source and a reference source are imaged using a transfer camera, one after the other, keeping all collection parameters the same. Using Eqs. (1) and (2) and rearranging terms, the radiance L(test) of the tissue phantom is determined.

### 2.2 Test and Reference Source

The tissue phantom test source used in this example is a 3D-printed polymeric structure with 1000 nM concentration of IR-125 and unspecified absorptance and scattering properties. A 4-mm-diameter calibrated aperture is affixed in front of it to define the area. First, the imaging parameters are established (focus, f-stop, and distance). The tissue phantom is then irradiated with <5-mW excitation radiation to generate the fluorescence, and the irradiance level is recorded prior to collecting the tissue phantom image. After the tissue phantom image collection, an image of the reference source is then taken. The radiant flux from the tissue phantom surface is captured by the camera lens and relayed through the lens aperture at the given f-stop, forming an image of the source at the detector plane.

**Fig. 3:**
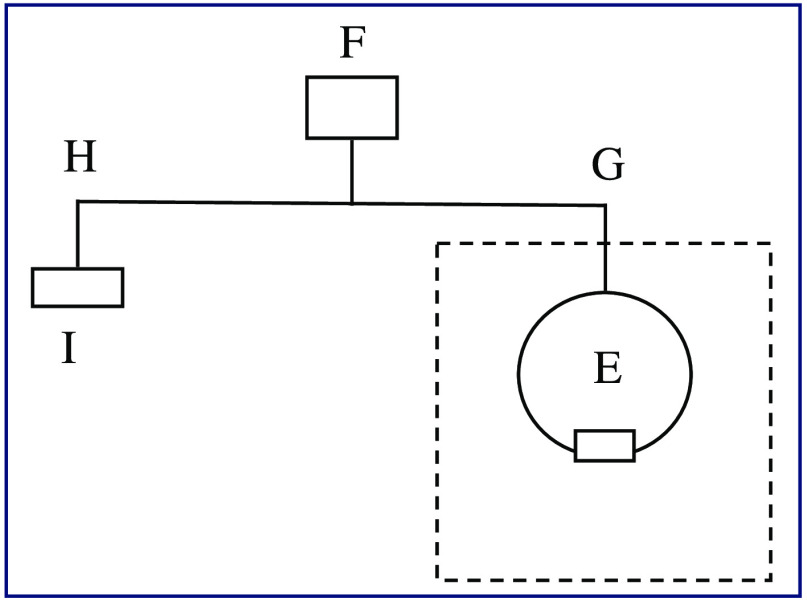
LED source (F) emission is split using a bifurcated fiber with the 90% branch (H) going to a photodiode monitor (I) and the 10% branch (G) input into the integrating sphere (E), which serves as the reference source.

Figure 3 shows a diagram of a measurement station set up for measuring a dim test source such as a tissue phantom or other low radiance sources. The inset is the portion shown in Fig. 2. The reference source is the exit port of a 50.8 mm (2-in.) diameter integrating sphere onto which the same aperture used for the tissue phantom is affixed. Optical radiation from an 850 nm LED (F) (M850F2, Thorlabs) is split using a bifurcated optical fiber (TM50R2S1B, Thorlabs) (G, H), with a 90:10 split ratio. Output from the 90% branch (H) is measured using another calibrated photodiode (S2281-04, Hamamatsu) (I) as a monitor; output from the 10% branch (G) is input into the integrating sphere (E). This is an updated version of the measurement method described in Ref. 3, with additional redundancy in SI-traceability and expected lower measurement uncertainty. It is currently under internal performance validation and uncertainty budget development prior to availability for calibration service.

Specific steps are taken to minimize contributions to the measurement uncertainty budget. To minimize any detector nonlinearity effects, the LED source (F) drive current is adjusted until the average counts observed from the image of the reference source match that from the tissue phantom. No changes are made to the camera settings between the test and reference source measurements. All measurements of reference and test sources are made with the source normal to the transfer camera or test imager and centered along the optic axis. As with all radiometric measurements, alignment and dimensional measurements are major contributors to the measurement uncertainty.^20^^,^^21^

### 2.3 Tissue Phantom Calibration Value

For a given tissue phantom preparation (specific fluorophore, absorber, and scatterer concentration), the tissue phantom is calibrated for radiance $L(\lambda_{\mathrm{em}})$ normalized to the excitation irradiance $E(\lambda_{\mathrm{ex}})$ at a specified angle of incidence. The character F is used here to express this ratio

$$F(\lambda_{\mathrm{em}},\lambda_{\mathrm{ex}})=\frac{L(\lambda_{\mathrm{em}}),}{E(\lambda_{\mathrm{ex}})},$$ (4)

where F is an aggregate fluorescence yield factor over the surface of the tissue phantom. It is called an aggregate fluorescence yield to distinguish it from the normally reported molar fluorescence yield of the pure fluorophore, which is an intrinsic optical property of the material. F includes modifying factors such as absorptance of the material at the excitation wavelength, the fluorescence yield, and the amount of fluorophore in the sample being irradiated with excitation radiation. At the time the tissue phantom is used to calibrate a test imager, $E(\lambda_{\mathrm{ex}})$ is measured at the object plane to find the corresponding tissue phantom radiance.

### 2.4 Example Calibration of a Tissue Phantom

In this example, the radiance of the test source (tissue phantom) is determined using Eq. (5). The reference source is calibrated using an NIST reference spectroradiometer^22^

$$\frac{S(\text{test})}{S(\mathrm{ref})}=\frac{L_{\text{source}}(\text{test})\cdot (\frac{A_{\text{lens apt}}}{d^{2}})\cdot A_{\text{source}}(\text{test})}{L_{\text{source}}(\mathrm{ref})\cdot (\frac{A_{\text{lens apt}}}{d^{2}})\cdot A_{\text{source}}(\mathrm{ref})}.$$ (5)

Table 2 shows an example of data from a tissue phantom radiance measurement against the reference source. S is the sum of counts from all pixels in the image ($N_{i}$) attributable to the radiant flux at the image, $\Phi_{\text{image}}$. The total counts are determined by drawing the circular regions of the images of the fluorescent sources using image processing software (ENVI, L3Harris Geospatial). Radiance of the same phantom affixed with a 4 and 5 mm aperture is shown in Table 2 along with the excitation irradiance used and the derived F value. Using Eq. (1), $\Phi_{\text{image}}$ is calculated. The solid angle ω is calculated using the area of the lens aperture (f1.4/23) at the f-stop used (f11; $A_{\text{lens apt}}$ is $0.034\;{\mathrm{cm}}^{2}$) and the distance from the sample to lens aperture (15.0 cm). The $R_{f}$ for the imager using radiance information is shown in the last column. This is the counts reported per mW of $\Phi_{\text{image}}$ detected.

**Table 2:** Example data on a tissue phantom calibrated for radiance against a reference source. The same phantom is measured with a 4- and 5-mm diameter aperture.

Source	$A_{\text{source}}$ (${\mathrm{cm}}^{2}$)	S	No. of pixels	$L_{\text{source}}$	$E(\lambda_{\mathrm{ex}})$	$F(\lambda_{\mathrm{em}},\lambda_{\mathrm{ex}})$	$\Phi_{\text{image}}$	$R_{f}$ (imager)
Reference	0.1256	$3.1\times 10^{7}$	1682	$4.10\times 10^{-3}$			$7.68\times 10^{-8}$	$3.95\times 10^{14}$
Test (4 mm)	0.1256	$3.0\times 10^{7}$	1682	$3.92\times 10^{-3}$	4.80	$8.16\times 10^{-4}$	$7.51\times 10^{-8}$	$3.95\times 10^{14}$
Test (5 mm)	0.1963	$4.9\times 10^{7}$	2476	$4.14\times 10^{-3}$	3.33	$1.24\times 10^{-3}$	$1.24\times 10^{-7}$	$3.95\times 10^{14}$

**Table 3:** Illustration of how the calibration value F of the tissue phantom can be used to estimate signal changes.

Calibration value	Value	Unit	Source	
F	$8.16\times 10^{-4}$	$\mathrm{mW}\;{\mathrm{cm}}^{-2}\;{\mathrm{sr}}^{-1}/\mathrm{mW}\;{\mathrm{cm}}^{-2}$	Previous calibration	Table 2
Excitation irradiance	1.40	$\mathrm{mW}\;{\mathrm{cm}}^{-2}$	Measured	At the point of use
Expected radiance	$1.14\times 10^{-3}$	$\mathrm{mW}\;{\mathrm{cm}}^{-2}{\mathrm{sr}}^{-1}$	Calculated	Eq. (4)
$R_{f}$ imager	$3.95\times 10^{14}$	$\text{counts }{\mathrm{mW}}^{-1}$	Previous calibration	Table 2
Lens f-stop	5.6	—	Experimental	At the point of use
Lens aperture area	0.13	${\mathrm{cm}}^{2}$	Calculated	f-stop and lens f
Source area	0.1256	${\mathrm{cm}}^{2}$	Previous calibration	Table 2
Distance	19.5	cm	Measured	At the point of use
Expected flux	$5.0\times 10^{8}$	mW	Calculated	Eq. (1)
Expected counts	$1.98\times 10^{7}$	Total counts S	Calculated	Eq. (3)
Measured counts	$1.96\times 10^{7}$	Total counts S	Measured	Image data

### 2.5 Use of the Imager Fluorescence Band Responsivity $R_{f}$

Changes to optical collection affect the imager signal. In this example, the now calibrated tissue phantom is imaged using the same camera at a different f-stop (5.6), at a different distance, at a different excitation irradiance, and on another day. The expected radiant flux and expected counts are calculated using the tissue phantom F and the imager $R_{f}$ values (Table 3).

### 2.6 Estimates of Uncertainty

Table 4 is a list of the successive steps in the determination of the $R_{f}$ of an imager using a tissue phantom calibrated for radiance. As shown in Fig. 1, each step in the calibration chain increases the measurement uncertainty. The procedure for the propagation of uncertainties according to Ref. 23 is followed.^23^ Relative standard uncertainty u of the measured quantity, expressed in % at coverage factor k=1, is generally used; expanded uncertainty U at coverage factor k=2 is noted wherever it is used. Tables with a nonexhaustive list of the contributors to uncertainty for each of these steps are shown in the Appendix.

**Table 4:** Successive steps in determining $R_{f}$ from a calibrated tissue phantom and the cumulative increase in its relative standard uncertainty.

Calibration step	Measured quantity	Relative standard uncertainty (%)
Tissue phantom calibration	L(test)	6.0
Radiance to excitation irradiance ratio	F	6.2
Determining image radiant flux	$\Phi_{\text{image}}$	8.4
Measuring imager response	$R_{f}$	8.4

### 2.7 Choice of Imager Settings for Calibrating an Imager

It is beneficial to calibrate the imager at all anticipated configurations; parameters include exposure times, aperture settings, distances, and angles. This is important for systems in which the optical configuration can be changed during use, such as an imager with a zoom lens on an articulating arm, and corrections are needed. The following is an illustration of the effects of the choice of imager setting when determining $R_{f}$ of an imager.

It should be noted that the measurements in this section were performed prior to the tissue phantom calibrations described in the previous section, to explore whether it is feasible to do an imager responsivity calibration *in situ* using a portable source such as an LED source or a tissue phantom.

**Fig. 4:**
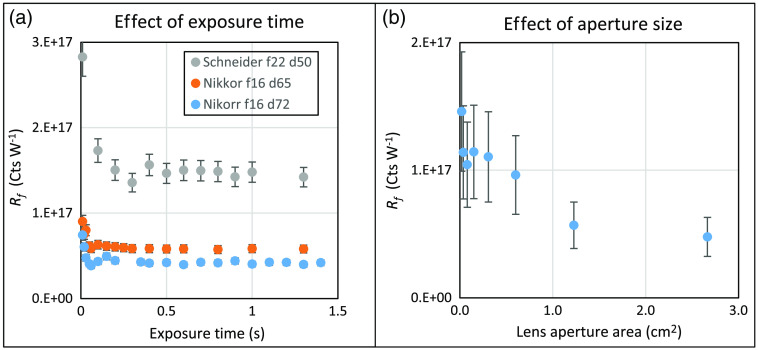
(a) The effect of exposure time on the imager response value taken at different distances with the smallest aperture for two lenses. (b) Larger aperture settings gave lower than predicted camera response due to vignetting by a filter mount installed in front of the lens and sensor saturation; these were subsequently verified by measurements.

The $R_{f}$ of a camera (a second Pixis 1024BR, Princeton Instruments) is measured using a source (850 nm LED, Thorlabs) of known radiance. The camera was fitted with two different lenses and used at three different distances. Figure 4(a) shows the effect of exposure time on the measured $R_{f}$ value for different distances and lenses at the smallest aperture setting, as this allowed for long exposure times without saturation. In Fig. 4(a), the uncertainty bars shown (U=8% at k=2) are from the relative standard uncertainty of data collected at exposure times 0.2 s and longer. Short exposure times can lead to large uncertainties because the variability in the time that it takes the shutter to open and close becomes a significant proportion of the exposure time. Figure 4(b) shows the camera response at various aperture settings of the Schneider lens at 50.0-cm distance for 1.0-s exposure time, obtained using a linear fit of the acquired data at various exposure times, since a 1.0-s exposure time is not uniformly feasible at all aperture settings. The $R_{f}$ at larger apertures were lower than expected due to vignetting by an optical filter mount placed in front of the camera lens, and sensor saturation, both of which were verified by measurements. At the smallest aperture setting, the signal-to-noise ratio is low, increasing the uncertainty. At the largest aperture setting, stray light due to reflectance and scattering from surfaces near the lens edge and aberrations at the outer edges of the lens can affect the collected radiant flux in unexpected ways, also increasing the uncertainty. Thus, for Fig. 4(b), relative standard uncertainty using all eight aperture settings is >30% and reduces to 7% when only the middle five aperture settings are used. Table 5 summarizes the different optical configurations used to determine mean $R_{f}$, excluding the smallest and largest apertures.

**Table 5:** Measurement of mean $R_{f}$ value across several different optical configurations using a calibrated radiance source.

Test No.	Lens	Distance (m)	$L_{\text{source}}$ $W\;m^{-2}\;{\mathrm{sr}}^{-1}$	No. of f-stops used/available	Mean $R_{f}$ (Cts $W^{-1}$)	$u(R_{f})$ (%)
1	Nikkor f1.4/50	0.65	0.045	6/8	7.5E16	12.6
2	Nikkor f1.4/50	0.72	0.045	6/8	6.0E16	5.0
3	Schneider f1.9/35	0.50	0.108	5/8	1.1E17	7.0

**Fig. 5:**
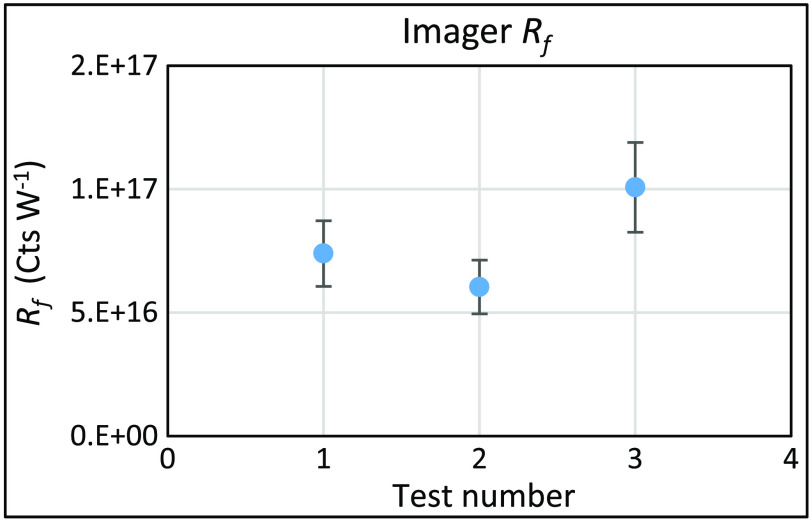
Imager $R_{f}$ at three different optical configurations shown with $U(R_{f})$ at 18% at (k=2).

This exercise shows that there are optimal settings at which to perform a calibration of the imager. In Fig. 5, the imager response $R_{f}$ at the three different sets of optical configurations from Table 5 is calculated using the radiance values from the calibrated source. For each test number, the $R_{f}$ is the mean value over measurements taken at various exposure times and over several f-stops. Using test data at all configurations shown in Table 5, the relative standard uncertainty in the mean $R_{f}$ value, $u(R_{f})$, is 9%. This is shown in Fig. 5 with expanded uncertainty U at 18% (k=2). The results were not corrected for the lens transmittance and likely account for the higher value measured with the Schneider lens, as it has a higher transmittance specification in the near-infrared region.

## 3 Summary

Tissue phantoms that are designed to monitor the repeatability of an optical imager’s fluorescence measurement can be calibrated to be SI-traceable and can subsequently be used to measure an optical imager’s responsivity to fluorescence for the specified spectral band. In general, tissue phantom composition and preparation can vary from one laboratory to another, or a set of tissue phantoms may be intentionally varied to represent specific tissue optical characteristics. Use of a standard SI-traceable tissue phantom removes the variance associated with unique phantoms used in each laboratory for a specific device. It is important to establish SI-traceability for the community to develop minimum specifications for an imager’s response to fluorescence.

Tissue phantoms that are designed for use as working standard sources for establishing SI-traceability need to exhibit photostability, i.e., the fluorescence emittance is sufficiently stable for a given period of time (e.g., 1 year), homogeneous, spatially uniform, and of simple geometric construction. The material composition of the tissue phantom to be calibrated must be specified because the optical properties, such as the concentration of absorbers and scatterers, influence the surface fluorescence emitted and subsequently measured.

In this paper, only the use of a tissue phantom as a working standard to calibrate an imager has been described. Because the tissue phantom is an optical radiation source, it can also be used as a reference for the contrast agent, for its calibration is independent of any specific imaging system. Thus, the same working standard can serve different parties with interest in the FGI community and allow for instrument-based validation such that adjustments and optimization can be made prior to regulatory review and preclinical and clinical studies.

## 4 Appendix: Radiometric Method to Calibrate an Imager Using Calibrated Tissue Phantoms

When the tissue phantom is calibrated by a calibration laboratory, the values are expressed as F, which is emission radiance per excitation irradiance [Eq. (1)]; this needs to be multiplied by the excitation irradiance at the point of use to yield the corresponding radiance of the source, $L_{\text{source}}$.

**Fig. 6:**
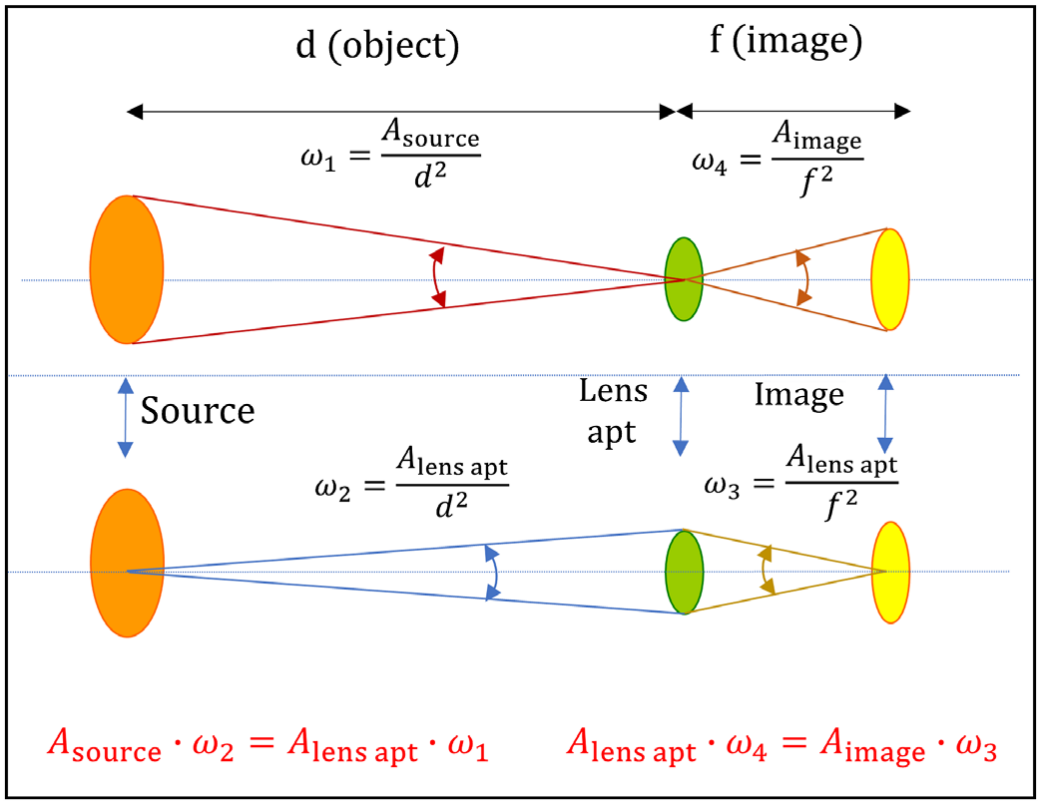
The product Aω for an optical system is a constant. This invariance allows for quantitative determination of the radiant flux from an extended source relayed through the lens aperture and projected onto the imager.

Figure 6 shows a simplified diagram of the radiometric quantities that apply to the calibration of the imager response using a tissue phantom as the calibration source. For illustration purposes, all surfaces are perfectly aligned with respect to the optic axis and are perpendicular to each other. In practice, surfaces are not perfectly flat and tilted with respect to the center lines, and thereby cosine corrections need to be applied. Energy is conserved as light propagates in free space, with the product of the area of a surface and the solid angle subtended being a constant, in either direction. Radiance is invariant; thus $L_{1}$ is equal to $L_{2}$ as

$$\Phi =L_{1}\omega_{1}A_{2}=L_{2}\omega_{2}A_{1}.$$ (6)

Equations (7)–(10) show the equations relating the radiant flux to radiance and the measurable dimensional quantities that define the optical measurement. The radiant flux passing through the lens aperture, $\Phi_{\text{lens apt}}$, is a function of the radiance of the source $L_{\text{source}}$; the solid angle of collection, $\omega_{\text{lens apt}}$, which is defined by the ratio of the lens aperture area to the square of the distance; and the area of the source, $A_{\text{source}}$. The radiant flux (reduced by the lens transmittance) is projected onto the sensor as an image, generating counts from pixels that comprise the image of the source

$$\Phi_{\text{lens apt}}=\Phi_{\text{image}},$$ (7)

$$\Phi_{\text{lens apt}}=L_{\text{source}}\omega_{\text{lens apt}}A_{\text{source}},$$ (8)

$$\omega_{\text{lens apt}}=\frac{A_{\text{lens apt}}}{d^{2}},$$ (9)

$$\Phi_{\text{image}}=L_{\text{source}}\frac{A_{\text{lens apt}}}{d^{2}}A_{\text{source}}.$$ (10)

Equation (10), which is also Eq. (1), shows the relationship between the radiant flux at the image and the radiance of the source.

Measuring the tissue phantoms over a range of radiances, e.g., by varying the excitation irradiance, yields the imager’s $R_{f}$ value(s) with respect to radiant flux. The $R_{f}$ is ideally constant (within estimated uncertainty) or a slowly varying function. This gives the user practical information on the imager’s operational dynamic range. The $R_{f}$ value can then be used to quantify the radiance of other sources such as contrast agents as fluorescence exits the surface and into free space.

A measurement result must have an accompanying stated uncertainty, an essential element of metrological traceability to the SI. Tables 6–9 show nonexhaustive lists of the contributors to uncertainty in the measurement values at each of the steps outlined in the use of a tissue phantom to determine the $R_{f}$ of an imager. These are only current estimates of their magnitude. As shown in Fig. 1, uncertainties increase with each succeeding comparison step. Dimensional measurements, especially the internal lens aperture area and distances, are significant contributors to the uncertainty. The challenge in the calibration laboratory is to reduce the uncertainty in the measurement of low radiance sources.

**Table 6:** Contributors to the relative standard uncertainty u(L(test)) in the radiance L(test) calibration of a tissue phantom against a reference radiance source L(ref).

Contributor	Estimation source	u(L(test)) (%)
S (test)	Std u of mean counts from image	0.6
S (ref)	Std u of mean counts from image	0.4
L (ref)	Spectroradiometer calibration	2.0
$A_{\text{lens apt}}$	Mfr. data	5.0
$d^{2}$	Measured	3.0
$A_{\text{source}}$	Measured (calibration lab)	0.1
$A_{\text{test}}$	Measured (calibration lab)	0.1
u(L(test)) (k=1)	—	6.2

**Table 7:** Contributors to the relative standard uncertainty $u(F(\lambda_{\mathrm{em}},\lambda_{\mathrm{ex}}))$.

Contributor	Estimation source	$u(F(\lambda_{\mathrm{em}},\lambda_{\mathrm{ex}}))$ (%)
L(test)	Table 6	6.2
$E(\lambda_{\mathrm{ex}})$	Measurement	0.5
$u(F(\lambda_{\mathrm{em}},\lambda_{\mathrm{ex}}))$ (k=1)	—	6.2

**Table 8:** Contributors to relative standard uncertainty in $u(\Phi_{\text{image}})$ using L(test) from a calibration.

Contributor	Estimation source	$u(\Phi_{\text{image}})$ (%)
L(test)	Table 6	6.2
Throughput	Measurement	0.5
$A_{\text{lens apt}}$	Mfr data	5.0
$d^{2}$	Measurement	3.0
$A_{\text{source}}$	Calibration	0.1
$u(\Phi_{\text{image}})$ (k=1)	—	8.4

**Table 9:** Contributors to relative standard uncertainty $u(R_{f})$.

Contributor	Estimation source	$u(R_{f})$ (%)
S (test)	Std u in pixel counts	0.6
$\Phi_{\text{image}}$	Table 8 (above)	8.4
$u(R_{f})$ (k=1)	—	8.4

## References

[1] Barth C. W. Gibbs S. L., “Fluorescence image-guided surgery-a perspective on contrast agent development,” Proc. SPIE 11222, 112220J (2020). PSISDG 0277-786X 10.1117/12.2545292 PMC7115043 32255887

[2] Koch M. Symvoulidis P. Ntziachristos V., “Tackling standardization in fluorescence molecular imaging,” Nat. Photonics 12, 505–515 (2018). NPAHBY 1749-4885 10.1038/s41566-018-0221-5

[3] Zhu B. et al., “Determining the performance of fluorescence molecular imaging devices using traceable working standards with SI units of radiance,” IEEE Trans. Med. Imaging 35(3), 802–811 (2016). ITMID4 0278-0062 10.1109/TMI.2015.2496898 26552078 PMC5304482

[4] Zhu B. Sevick E. Litorja M., “Comparison of NIR versus SWIR fluorescence imaging of indocyanine green using SI-derived metrics of image performance,” IEEE Trans. Med. Imaging, 39(4), 944–951 (2020). ITMID4 0278-0062 10.1109/TMI.2019.2937760 31478842

[5] “International vocabulary of metrology—basic and general concepts and associated terms,” JCGM 200:2008, VIM 3rd ed. (2008). 10.1016/j.clinbiochem.2008.09.007 19863914

[6] “Reference materials-selected terms and definitions,” ISO Guide 30 (2015).

[7] Joint BIPM, OIML, ILAC and ISO, “Declaration on metrological traceability” (2018).

[8] Pogue B. W. Patterson M. S., “Review of tissue simulating phantoms for optical spectroscopy, imaging and dosimetry,” J. Biomed. Opt. 11(4), 041102 (2006). JBOPFO 1083-3668 10.1117/1.2335429 16965130

[r9] Anastasopoulou M. et al., “Comprehensive Phantom for interventional fluorescence molecular imaging,” J. Biomed. Opt. 21(9), 091309 (2016). JBOPFO 1083-3668 10.1117/1.JBO.21.9.091309 27304578

[10] Gorpas D. et al., “Benchmarking of fluorescence cameras through the use of a composite phantom,” J. Biomed. Opt. 22(1) 016009 (2017). JBOPFO 1083-3668 10.1117/1.JBO.22.1.016009 28301638

[11] Kanniyappan U. et al., “Performance test methods for near-infrared fluorescence imaging,” Med. Phys. 47(8), 3389–3401 (2020). 10.1002/mp.14189 32304583 PMC7496362

[12] Ruiz A. J. et al., “Indocyanine green matching phantom for fluorescence-guided surgery imaging system characterization and performance assessment,” J. Biomed. Opt. 25(5), 056003 (2020). JBOPFO 1083-3668 10.1117/1.JBO.25.5.056003 PMC7240319 32441066

[13] Liu Y. et al., “Biomimetic 3D-printed neurovascular tissue phantoms for near infrared fluorescence imaging,” Biomed. Opt. Express 9(6), 2810–2824 (2018). BOEICL 2156-7085 10.1364/BOE.9.002810 30258692 PMC6154206

[14] Wang L. DeRose P. Gaigalas A. K., “Assignment of the number of equivalent reference fluorophores to dyed microspheres,” J. Res. NIST 121, 264–280 (2016). 10.6028/jres.121.012 PMC7351568 34434623

[15] “Certificate of analysis for standard reference material 1934: fluorescent dyes for quantitative flow cytometry,” https://www-s.nist.gov/srmors/certificates/1934.pdf.

[16] “Flow cytometry standards consortium,” Fed. Reg. Notice 85 FR 64444, 2020, Federal Register: The Daily Journal of the United States, https://www.federalregister.gov/documents/2020/10/13/2020-22620/flow-cytometry-standards-consortium.

[17] Ruiz A., “Quel imaging,” White River Junction, Vermont; fabricated tissue phantoms used in this measurement example.

[18] References are made to certain commercially available products in this paper to adequately specify the experimental procedures involved. Such identification does not imply recommendation or endorsement by the National Institute of Standards and Technology, nor does it imply that these products are the best for the purpose specified.

[19] Larason T. C. Houston J. M., “NIST measurement services: spectroradiometric detector measurements: ultraviolet, visible and near-infrared detectors for spectral power,” NIST Special Publications 250-41 (2008).

[20] Nicodemus F. E., Self-Study Manual on Optical Radiation Measurements-Part 1: Concepts, Vol. 1, US Dept. of Commerce Nat. Bureau of Standard, Washington (1976).

[21] Litorja M. et al., “Lambertian nature of tissue phantoms for use as calibrators in near infrared fluorescence imaging,” Proc. SPIE 9696, 96960H (2016). PSISDG 0277-786X 10.1117/12.2216324

[22] Zong Y. Ohno Y., “Realization of total spectral radiant flux scale and calibration service at NIST,” in Proc. 26th Session CIE, Beijing, D2-179-D2-182 (2007).

[23] Taylor B. N. Kuyatt C. E., “Guidelines for evaluating and expressing the uncertainty of NIST measurement results,” NIST TN 1297, 1994, https://www.nist.gov/pml/nist-technical-note-1297.

